# Enhancing activation in the right temporoparietal junction using theta-burst stimulation: Disambiguating between two hypotheses of top-down control of behavioral mimicry

**DOI:** 10.1371/journal.pone.0211279

**Published:** 2019-01-25

**Authors:** Korrina A. Duffy, Bruce Luber, R. Alison Adcock, Tanya L. Chartrand

**Affiliations:** 1 Department of Psychology and Neuroscience, Duke University, Durham, North Carolina, United States of America; 2 Department of Psychiatry and Behavioral Sciences, Duke University, Durham, North Carolina, United States of America; 3 Department of Neurobiology, Duke University, Durham, North Carolina, United States of America; 4 Fuqua School of Business, Duke University, Durham, North Carolina, United States of America; Shanghai Mental Health Center, CHINA

## Abstract

Whereas previous research has focused on the role of the rTPJ when consciously inhibiting mimicry, we test the role of the rTPJ on mimicry within a social interaction, during which mimicking occurs nonconsciously. We wanted to determine whether higher rTPJ activation always inhibits the tendency to imitate (regardless of the context) or whether it facilitates mimicry during social interactions (when mimicking is an adaptive response). Participants received either active or sham intermittent theta-burst stimulation (iTBS: a type of stimulation that increases cortical activation) to the rTPJ. Next, we measured how much participants mimicked the hair and face touching of another person. Participants in the active stimulation condition engaged in significantly less mimicry than those in the sham stimulation condition. This finding suggests that even in a context in which mimicking is adaptive, rTPJ inhibits mimicry rather than facilitating it, supporting the hypothesis that rTPJ enhances representations of self over other regardless of the goals within a given context.

## Introduction

Mimicry occurs in nearly every social interaction–from mimicking an interaction partner’s postures and gestures to facial expressions and verbal patterns. Even though the mimicker and the mimicked are generally not aware of its occurrence, mimicry has been shown to increase liking of the mimicker [1], enhance the smoothness of the interaction [1], increase feelings of affiliation, trust, and rapport [2],[3], and facilitate prosocial behavior [4]. Given the functional role that mimicry plays in social interactions, it makes sense that goals affect mimicry.

Affiliation goals, for example, have a particularly powerful effect on mimicry. The presence of an affiliation goal has been shown to increase mimicry within a social interaction [2]. This is taken to mean that mimicry can serve as a nonconscious strategy for affiliation and that affiliation goals lead people to engage in behaviors (including mimicry of others) that accomplish that goal. Affiliation goals that are triggered indirectly can also increase mimicry, such as affiliation goals that are triggered by the social context. For example, social exclusion powerfully motivates reaffiliation; participants who are socially excluded mimic more in a subsequent interaction than do those who are socially accepted prior to the interaction [5].

Romantic attraction can also serve as a powerful affiliation goal. Research suggests that heterosexual men mimic a physically attractive woman more when they are trying to express romantic interest [6],[7]. Interestingly, this effect is moderated by relationship status. Those men involved in romantic relationships, who presumably are not trying to affiliate with an attractive woman, mimic less than those who are not in a relationship. Furthermore, this effect in romantically-involved men is moderated by their reported closeness to [7] and love for [6] their romantic partner. Therefore, those with a presumed goal to express romantic interest mimic more than those who are happily coupled.

Previous research, then, converges on the finding that people mimic in a goal-directed, context-dependent way. This implies top-down control of mimicry. However, mimicry can also arise from bottom-up processes, as evidenced by its presence when affiliation goals are absent. Researchers have referred to a “perception-behavior link” to describe the phenomenon in which merely seeing someone engage in a behavior leads one to automatically enact the same behavior [1]. Evidence of a mirror system suggests on a neural level how bottom-up processes might lead to mimicry. Mirror neurons are neurons that are active both during action production (self) and action perception (other) [8],[9]. During action production and perception, motor representations, which are patterns of neural firing, are activated. Although the motor representations of action production and action perception are not identical, they overlap within the same neural system, sharing a subset of neurons [8],[9]. This neural system of shared motor representations of self and other blurs the boundary between action and perception, self and other [9]. Shared representations can explain the tendency for people to mimic one another; however, they do not explain why we do not mimic everything that we see others do. Because activation of the mirror neuron system does not always lead to mimicry, this implies top-down modulation of this bottom-up source of mimicry.

### Neuroscience perspective on the top-down control of mimicry

Researchers in the field of neuroscience have also examined top-down control of mimicry, albeit from a different angle than psychologists. Unlike psychologists, who have focused on how goals adaptively modulate mimicry, neuroscientists have concentrated on identifying which brain regions are involved in top-down control of mimicry. From the neuroscience perspective, a top-down control mechanism is necessary in order to distinguish between self-generated and other-generated motor representations, which otherwise could be confused due to a neural system of shared representations that gives rise to mimicry. Neuroscience evidence from fMRI studies suggests that top-down control of mimicry involves the right temporoparietal junction (rTPJ) [10],[11], but does not establish its precise role.

While the exact functions performed by rTPJ remain elusive, it is nevertheless a critical brain region underlying disorders that show dysregulated self-other processing, such as autism [12], borderline personality disorder [13], antisocial personality disorder [14], schizophrenia [15], mirror-touch synaethesia [16], Tourette syndrome [17], anorexia nervosa [18], and bulimia nervosa [19]. Many of these disorders also show atypical mimicry (autism: [20]; borderline personality disorder: [21]; schizophrenia: [22]; mirror-touch synaesthesia: [23]; Tourette syndrome: [24]). Although researchers that have used neurostimulation on healthy volunteers have suggested that neurostimulation could be therapeutic in disorders that show dysregulated self-other processing [25], only one study, to our knowledge, has actually tested and demonstrated its effectiveness (depersonalization disorder: [26]). In order to understand how rTPJ neurostimulation could be therapeutic in clinical populations, an important first step is understanding how exactly the rTPJ affects self-other processing and, by extension, its more measurable behavioral corollary–mimicry.

A challenge for researchers exploring the relationship between rTPJ activation and mimicry is that, in the scanner, participants have very limited mobility and any head movements greatly affect the quality of the brain scan. Thus, in order to study the control of imitation in a scanner, researchers have used a paradigm in which participants perform instructed finger movements while observing finger movements that are either congruent with their instructions (imitation) or incongruent with their instructions (imitation inhibition). The incongruent trials require participants to inhibit the automatic tendency to imitate observed movements. In order to successfully inhibit imitation, the participant must be able to distinguish between motor representations evoked by the self versus another. When this ability is enhanced, the tendency to imitate incongruent finger movements is reduced, resulting in faster performance of the instructed finger movement.

The paradigm described above is used in the following studies. One of the first studies that implicated the rTPJ in the control of imitation was an fMRI study designed to test whether the control of imitation relies on the same brain regions as the control of dominant response tendencies–for example, the Stroop task [10]. The results indicated that while inhibiting a dominant response was associated with higher activation in the prefrontal cortex, inhibiting an imitative response was associated with higher activation in brain regions involved in distinguishing self from other, including the rTPJ [10]. Other fMRI research has corroborated these findings [11]. Together, these studies show a correlation between rTPJ activation and control of imitation, suggesting that rTPJ is involved in top-down control of mimicry, enabling us to not automatically imitate all actions we observe others perform.

This promising correlational fMRI evidence led researchers to investigate whether the rTPJ plays a causal role in the control of imitation. Using different neuromodulation techniques, two studies have shown that neuromodulation of the rTPJ affects imitative control [25],[27]. In a transcranial direct current stimulation (tDCS) study, anodal stimulation (which increases overall neural excitation) of the rTPJ led to improved control of imitation [25]. Further evidence corroborating this finding comes from a study using repetitive transcranial magnetic stimulation (rTMS) [27]. In this study, 1 Hz stimulation (which lowers cortical excitability and leads to decreased activation) of the rTPJ resulted in poorer control of imitation. This was indicated by slower response times for incongruent trials compared to congruent trials when participants received rTMS to the rTPJ compared with a control site. Overall, these studies showed that inhibitory stimulation (administered via TMS) impairs imitative control, leading to more unintentional mimicry, whereas excitatory stimulation (administered via tDCS) enhances imitative control, leading to less unintentional mimicry.

### How does the right temporoparietal junction modulate mimicry?

Despite these intriguing findings, the mechanism underlying rTPJ modulation of mimicry remains unknown. The literature offers two hypotheses for how the rTPJ modulates mimicry. The first hypothesis suggests that rTPJ activation affects the overlap of self-other representations by increasing the distinction between oneself and others [11],[28]. The assumption in this hypothesis is that as the distinction between oneself and another increases, motor representation of self will be enhanced over motor representations of other, meaning that it will be easier to enact self-generated motor plans and ignore other-generated motor patterns. According to this hypothesis, higher activation in the rTPJ should lead to less mimicry in social interactions.

The second hypothesis suggests that the rTPJ affects control of self-other representations such that higher activation increases control, allowing people to switch between representations of self and others and to enhance either the representation of self-over-other or other-over-self in a flexible, adaptive, and goal-directed manner [29]. According to this hypothesis, higher activation in the rTPJ should lead to more mimicry in social interactions because mimicking in a social context is typically beneficial.

Thus far, most studies, which in general have employed the imitation inhibition paradigm, have not sought to distinguish between these two hypotheses. It should be pointed out that even if one wanted to, these hypotheses cannot be pitted against one another using the imitation inhibition paradigm because, in this paradigm, both hypotheses make the same prediction. In the imitation inhibition paradigm, higher rTPJ activation leads to less automatic imitation, which is required for successful performance on the task. This outcome could be explained by either hypothesis. According to the first hypothesis, higher rTPJ activation would decrease the automatic tendency to imitate by increasing the distinction between oneself and others, enhancing representations of self over other. According to the second hypothesis, higher rTPJ activation would decrease the automatic tendency to imitate by increasing control of self-other representations, enhancing the representation of self over other in an adaptive, goal-directed manner in order to perform successfully at the task.

A true test of these hypotheses would pit an adaptive response against a non-adaptive response. For example, mimicry occurring within a social interaction is adaptive and requires enhancement of representations of other over self. According to the first hypothesis, if higher rTPJ activation simply increases the self-other distinction, enhancing representations of self over other, then higher rTPJ activation should lead to *less* mimicry in a social interaction. According to the second hypothesis, however, if higher rTPJ activation flexibly controls self-other representations in a goal-directed manner, then representations of other should be enhanced over representations of self and higher rTPJ activation should lead to *more* mimicry since mimicry serves an adaptive function in social interactions.

Only one study has attempted to test these competing hypotheses [30]. In this study, researchers increased activation in either the rTPJ or the right inferior frontal cortex (rIFC) using tDCS before participants engaged in three tasks: (1) a social interaction task in which mimicking is an adaptive behavior, (2) an imitation inhibition task in which imitation needs to be inhibited for successful performance, and (3) a non-imitative control task. The results showed that, relative to a sham control, increased activation in the rIFC led to greater imitative control in the imitation inhibition task as well as higher mimicry in the social interaction task [30]. In contrast, increased activation in the rTPJ led to greater imitative control in the inhibition task but did not affect mimicry in the social interaction task. However, it is difficult to determine whether the null result for rTPJ reflected the absence of an effect or was due to low statistical power to detect an effect (which could have been due to tDCS being less spatially precise and having a weaker effect than other neuromodulatory methods). For this reason, we test the effect of rTPJ activation on mimicry using a type of rTMS called theta-burst stimulation (TBS), which is a form of patterned stimulation linked to potent neuromodulation in animal research and which has been shown to produce effects in humans that are stronger and longer lasting than standard rTMS protocols [31]. Furthermore, TMS is more precise than standard tDCS because it has better spatial resolution and the ability to target stimulation sites using neuronavigation. Better spatial resolution provides more precision in stimulating a desired brain region without inadvertently stimulating the surrounding cortex. Furthermore, the functional brain region of interest was determined using MNI coordinates derived from previous fMRI studies [10],[11],[32]. The MNI coordinates corresponded to the brain coordinates showing peak activation (averaged across the group) during tasks that involved the control of imitation. Thus, the functional region of interest was localized by applying these MNI coordinates to each participant’s brain scan. Furthermore, we were able to target the specific region of interest using neuronavigation, a computer-assisted technology that spatially maps a participant’s brain scan onto their head using cranial markers, allowing for greater precision in stimulating a target brain region.

### Study design

**Fig 1:**
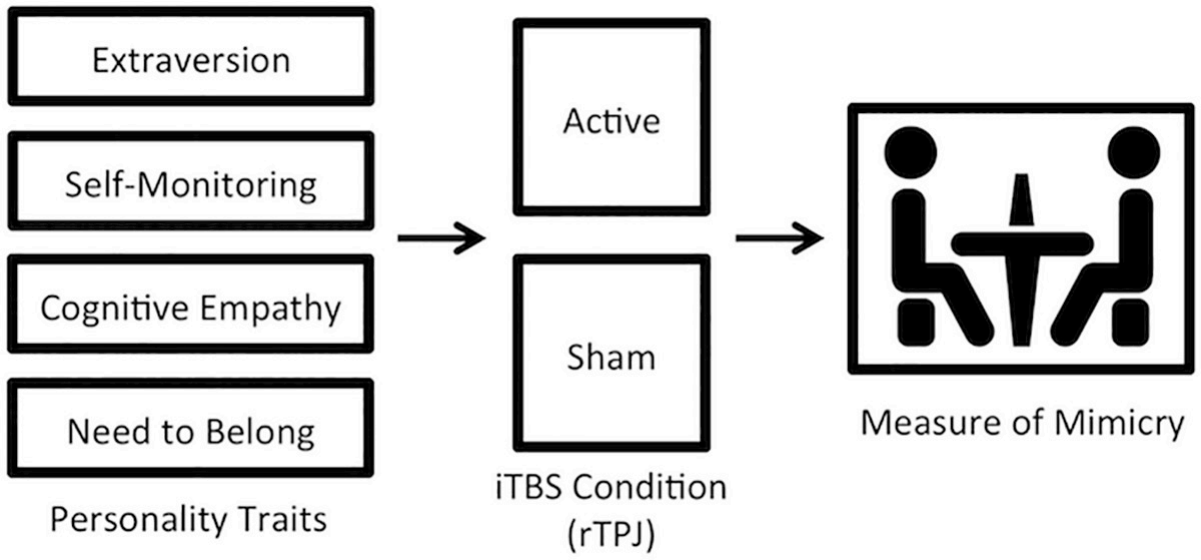
Schematic diagram of experimental procedure. Before the study session, participants completed an online questionnaire assessing demographic information as well as measuring personality traits that have been known to predict mimicry. When participants arrived for the study session, they were randomized to receive either active stimulation or sham stimulation to the right temporoparietal junction (rTPJ). Immediately following stimulation, participants interacted with a confederate in a 10-minute task that involved describing photos to one another. While they interacted, the confederate constantly touched her hair and face. The amount of time that participants touched their hair and face was measured by filming participants before the confederate was brought in (baseline) as well at while interacting with the confederate. The ratio of time spent touching hair and face during the social interaction relative to baseline was computed as a measure of how much the participant mimicked the confederate.

To disambiguate between these two competing hypotheses regarding the role of the rTPJ, participants were randomized to receive either active or sham intermittent TBS (iTBS: an excitatory pattern of stimulation) to the rTPJ in a between-subjects design. Next, we measured how much participants mimicked the hair and face touching of their social interaction partner. See Fig 1.

## Methods

### Participants

Seventy-two right-handed participants (20 males, 52 females) ages 18–40 (M = 24.4, SD = 5.0) were recruited from fMRI study subject pools. These subject pools were targeted because participants were eligible for the study only if they already had a structural brain scan. Given the social context of the study, we limited our sample age range to 18–40 years old because we wanted participants to be relatively similar in age to the confederate with whom they would be interacting. Participants in the subject pool were notified by email about the study. If participants were interested, they were screened on the phone to determine eligibility. When participants arrived at the lab, they signed written consent forms. They were then screened for any contraindications to the use of TMS. After being screened, participants provided a urine sample for pregnancy testing (if female) and drug testing to screen for drugs or medications that could increase the risks of seizure during TMS. Before and after iTBS, participants completed a measure of mood. After iTBS, potential side effects were assessed. At the end of the study, participants were thoroughly debriefed and given the opportunity to release their video data. Participants then completed payment forms and were mailed checks as compensation for their time. The study received approval by the Duke Medicine Institutional Review Board (IRB #Pro00053022).

Out of 71 participants, there were six reports of mild headaches, six reports of mild neck pain, three reports of mild to moderate scalp pain, three reports of mild sleepiness, two reports of mild trouble concentrating, and one report of mild memory problems. Regarding whether participants thought that the reported side effects were related to the TMS, of the headaches reports, two of the six participants thought that the relationship between the headache and TMS was “probable” while the other four thought it was “remote” to “possible;” of the neck pain reports, three of the six participants thought that the relationship between the neck pain and TMS was “none” to “remote” while the other three thought the neck pain was related to the posture of the chair; of the scalp pain reports, two of the three participants thought that the relationship between the scalp pain and the TMS was “definite” while one of the participants thought it was caused by the headband used to attach the tracker; of the sleepiness reports, all three of the participants thought that the relationship between the sleepiness and TMS was “remote” to “possible;” of the reports of trouble concentrating, one of the two participants thought that the relationship between having trouble concentrating and TMS was “remote” while the other participant thought that it was due to sleepiness; for the report of memory problems, the participant thought the relationship between the memory problems and TMS was “possible”.

Initially, we were testing an additional hypothesis about whether our results would be moderated by the presence of an affiliation goal. For fifteen participants, we attempted to manipulate an affiliation goal by telling the participant (before they were introduced to the confederate for the social interaction) that the next task had the best results when both people got along. However, our manipulation failed to increase affiliation motivation as intended. For this reason, we have excluded these 15 participants from our sample. In addition, one participant failed the drug test and therefore did not complete the study, leading to a final sample of 56 participants (19 males, 37 females; age, M = 24.6, SD = 5.3). The data that support the findings of this study are openly available within Open Science Framework at osf.io/5brqm. The protocol for the study are openly available at dx.doi.org/10.17504/protocols.io.wpvfdn6.

### Procedure

#### Personality traits

Participants were sent an email with a link to a questionnaire to complete before arriving for their study session. Studies have shown that mimicry is related to individual differences on personality traits such as extraversion [33], self-monitoring [34], perspective-taking [1], and the need to belong [35]. Thus, the initial questionnaire included scales that have correlated with mimicry: Goldberg’s Mini-Markers [36], Eighteen-Item Measure of Self-Monitoring [37], Perspective-Taking Subscale of Interpersonal Reactivity Index [38], and Need to Belong Scale [39]. Scores on these scales were assessed as potential covariates of mimicry. The questionnaire also gathered data on age, race, and handedness.

#### Motor threshold

As recommended by safety guidelines, motor threshold was used to determine the intensity of stimulation for each participant. Resting motor threshold was determined using right hemisphere motor cortex. Motor thresholds (and theta-burst stimulation) were performed using a MagPro X100 device (MagVenture, Denmark). The coil used in all stimulation procedures was the Cool-B65 Butterfly coil, with an outer diameter of 75mm (MagVenture, Denmark). All TMS pulses were biphasic throughout. In order to determine the motor threshold (MT), motor evoked potentials were measured from the first dorsal interosseous muscle using electromyography (EMG). The scalp region producing motor evoked potentials (MEPs) with the largest amplitude was identified and visually confirmed by movement of the index finger. Once the correct scalp region was identified, resting motor threshold was estimated using the Parameter Estimation by Sequential Testing (PEST), a computerized algorithm that uses a maximum-likelihood strategy [40]. The PEST procedure determines the minimum single-pulse output intensity necessary to elicit an MEP that has a peak-to-peak amplitude of ≥ 50μV (i.e., the resting motor threshold). After resting motor threshold was identified, active motor threshold was calculated using the formula: resting motor threshold = 120% active motor threshold [41].

#### Theta-burst stimulation

Brainsight software provided precise MRI-guided TMS coil placement on the scalp to target rTPJ. Prior to the study, structural MRI scans were manually registered to the standard MNI-152 template in the Brainsight neuronavigation system (Rogue Research, Montreal, Canada). The MNI coordinates used to localize rTPJ were based on a previous TMS study [27] that derived these coordinates from the average of the peak coordinates found by Brass and colleagues [10],[11] as well as Spengler and colleagues [32] when investigating the control of imitation. The rTPJ was targeted using MNI coordinates 54, −47, 26. These coordinates were fit to a corresponding point in the individual’s structural MRI, and the appropriate trajectory of stimulation to the surface of the head was set for each participant from this point, marking the location on the surface reconstruction where the center of the TMS coil would be placed.

Participants were randomized to receive either sham or active iTBS. The iTBS protocol consisted of trains of three pulses of TMS at 50 Hz beginning every 200 ms (i.e., at 5 Hz) and delivered at 80% of active MT [31]. There were 20 2 s trains, delivered beginning every 10 s for a total stimulation period of 192 s (a total of 600 pulses). All the procedures for active and sham stimulation were the same except that for sham stimulation, the coil was flipped 180 degrees on a vertical axis so that the scalp was shielded from the magnetic field output while retaining the auditory and some of the tactile aspects of active stimulation.

#### Measure of behavioral mimicry

Immediately after receiving either sham or active iTBS, participants were moved to a table with two chairs at the back of the room. As the experimenter moved the participant to a designated chair, the participant was told: “If you’d like to have a seat here then I will go ahead and get the other participant and explain the task to both of you when I get back.” The participant was then left alone in the room while the experimenter went to get the confederate. During this time alone in the room, the participant was filmed with a hidden camera to establish a baseline for amount of time hair and face touching. The confederate was then brought into the room and the experimenter explained the task, which involved taking turns describing photos [1]. A flip board in between the participant and confederate held the photos such that only the person whose turn it was to describe the photo could see the photo. They were told to describe the photo for a minute until a timer beeped and then they would switch roles. The photos were interesting scenes that would be relatively easy to describe for one minute (e.g. a family portrait in which each person is sitting on a giant lily pad in a pond). While the participant and the confederate took turns describing photos, the confederate touched her hair and face in various subtle ways. The participant was filmed during the 10-minute social interaction. The videos were later coded for how much time participants touched their hair and face while alone and during the social interaction. For each participant, we computed a ratio of time touching hair and face when alone in the room versus when interacting with the confederate. Because the confederate was instructed to touch her hair and face almost constantly, the amount of time the confederate touched her hair and face were not calculated to be used as a potential covariate. For consistency, only the first 30 s were coded of the time that participants were alone in the room.

#### Funneled debriefing

Suspicion regarding the experimental manipulations was probed in a questionnaire that asked participants what they thought the purpose of the study was. Along these lines, participants rated whether or not they thought that iTBS had affected their behavior; whether or not they noticed the confederate engaging in any particular gestures and mannerisms; whether or not they noticed themselves engaging in any of these same gestures and mannerisms; whether or not they thought they received active versus sham iTBS; and how confident they felt about their answer (1 = *very unconfident* to 5 = *very confident*). We used these items to test whether awareness of mannerisms had been affected by experiemental condition (sham vs. active iTBS).

In order to measure affiliation within the social interaction, we asked participants to rate how motivated they were to get along with the confederate (1 = *not at all* to 5 = *very much*); how much effort they made to get along with the confederate (1 = *none* to 5 = *a lot*); and how much they thought the confederate liked them (1 = *not at all* to 5 = *very much*). Given that responses on these three items hung together (alpha = .69) and all seemed to tap affiliation, we averaged the three items to create a composite of affiliation-related psychological processes. We used the composite score to test whether affiliation-related psychological processes had been affected by experimental condition (sham vs. active iTBS).

## Results

### Statistical analyses

All analyses were conducted using SPSS (version 21). Due to the participant exclusions that we previously mentioned, we had a final overall sample of 56 participants. However, additional data was missing for mimicry and personality traits so the sample size varied depending whether those variables were in the analysis. For analyses using mimicry, the sample size was 47 participants. In order to compute our measure of mimicry, we divided hair and face touching during the task by hair and face touching when alone. For eight participants, we could not compute a ratio because they did not touch their hair and face during the baseline (leading to a zero in the denominator). To correct for normality violations, we then log-transformed this ratio of hair and face touching (after transformation: skew = 0.06; kurtosis = -1.01). For one participant, we could not perform a log-transformation because the participant did not touch their hair and face at all during the task or baseline (leading to log-transformation of zero). Thus, after these nine participants were excluded, we had 47 participants with valid ratios on our measure of mimicry. For analyses using personality traits (extraversion, self-monitoring, need to belong, and perspective-taking), the sample size was 55 participants due to one participant not completing the personality trait scales.

### Does iTBS of the rTPJ affect behavioral mimicry?

**Fig 2:**
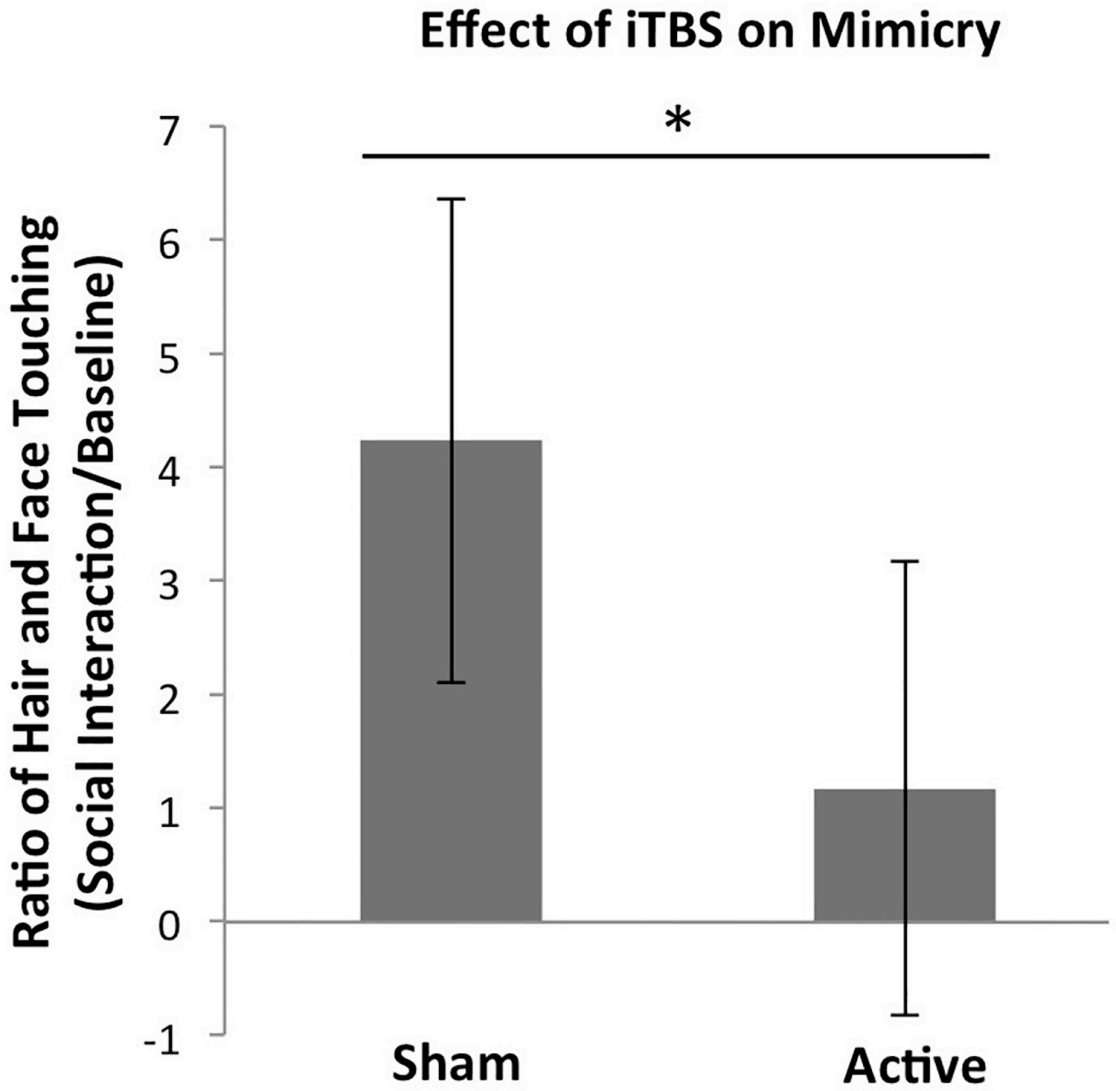
The effect of experimental condition (sham vs. active iTBS) on mimicry (ratio of hair and face touching during the social interaction versus when alone). Relative to those who received sham iTBS, those who received active iTBS mimicked the confederate significantly less. This suggests that active iTBS increases the distinction between self and other resulting in less behavioral mimicry. Error bars represent 95% confidence intervals.

In order to address our key question of whether our experimental condition (sham vs. active iTBS) affected how much participants mimicked the confederate during the social interaction, an ANCOVA was conducted with experimental condition as a between-subjects factor, confederate as a covariate (dummy-coded for the two confederates), and our measure of mimicry (log-transformed hair and face touching ratio) as the dependent variable. Even though we selected two confederates for the study who were both likeable undergraduate women, participants nevertheless mimicked one confederate more than the other. Therefore, we included confederate as a covariate in our model in order to control for this effect. As a reminder, for this test, we had 47 participants. The effect of experimental condition on mimicry was significant: F (1, 44) = 6.29, p = .02, η^2^ = .11, such that active iTBS (M = 1.17, SD = 2.71) led to lower mimicry than sham iTBS (M = 4.23, SD = 6.72). We controlled for confederate because it significantly explained variance in mimicry, F (1, 44) = 9.40, p = .004, η^2^ = .16. Although all analyses were performed on the log-transformed ratio of hair and face touching, for interpretability, all figures as well as means and standard deviations are based on raw values of the ratio of hair and face touching. See Fig 2.

### Is this effect mediated by a motivation to affiliate?

In order to test whether the effect of experimental condition (sham vs. active iTBS) on mimicry is mediated by affiliation-related psychological processes (i.e., whether iTBS affects affiliation motivation), we tested whether experimental condition resulted in differences on the composite score of affiliation-related psychological variables. In order to assess this, we ran an independent samples t-test in order to compare means on the affiliation composite score in the sham and active iTBS conditions. For these analyses, the sample size was 56 participants. There was not a significant difference in the affiliation composite score in the sham iTBS (M = 3.83, SD = 0.59) and active iTBS (M = 3.93, SD = 0.67) conditions, t (54) = -0.62, p = .54, 95% CI [-0.44, 0.23]. This suggests that the experimental condition did not affect affiliation-related psychological processes. Therefore, motivation to affiliate is not a potential mediator of our key finding.

### Do personality traits predict behavioral mimicry?

Next, in order to address whether personality traits (extraversion, self-monitoring, need-to-belong, and perspective-taking) should be included as covariates in our ANCOVA model discussed above, we ran bivariate correlations between mimicry (log-transformed hair and face touching ratio) and each of these personality trait variables. Any personality traits significantly correlated with mimicry would be included as covariates in our ANCOVA model. As a reminder, for these analyses, the sample size was 47 participants. None of the personality trait variables were significantly correlated with mimicry (extraversion: r = .04, p = .78; self-monitoring: r = .16, p = .28; need to belong: r = -.16, p = .28; perspective-taking: r = -.12, p = .43). This suggests that the personality traits that we measured do not explain variance in mimicry. Therefore, we do not include any of the personality traits as covariates in our ANCOVA model.

### Can our findings be explained by confounds?

Finally, we tested for potential confounds. First, we tested whether participants were successfully randomized to our experimental condition (sham vs. active iTBS). In order to assess this, we ran four separate independent samples t-tests comparing the means of our personality trait variables (which were assessed before participants came to the lab) in the sham iTBS and active iTBS conditions. Any significant effects would indicate that our procedure failed to randomize and we would test whether this effect explained our key finding by including that personality trait as a covariate in the ANCOVA model explained above. As a reminder, for these analyses the sample size was 55 participants. We found that experimental condition did not significantly predict extraversion, t (49.57) = -0.78, p = .44 (degrees of freedom adjusted due to a significant Levene’s Test for Equality of Variances), self-monitoring, t (53) = -0.26, p = .80, and need to belong, t (53), p = .45, suggesting successful randomization for these personality trait variables. However, experimental condition did significantly predict perspective-taking (sham iTBS: M = 3.43, SD = 0.66; active iTBS: M = 3.80, SD = 0.58), t (53) = -2.185, p = .03, suggesting failure to randomize for this personality trait variable. Despite the fact that higher perspective-taking has been associated with more mimicking in social interactions [1] and the active iTBS condition has higher perspective-taking and less mimicking, we still tested for potential confounding. In order to do this, we included perspective-taking in the ANCOVA model described above. As a reminder, for this analysis, the sample size was 47. The effect of experimental condition (sham vs. active iTBS) on mimicry (log-transformed hair and face touching ratio) remained significant, F (1, 43) = 5.62, p = .02, and the effect of perspective-taking did not significantly account for variance in mimicry, F = (1, 43) = 0.09, p = .77. Therefore, perspective-taking did not appear to be driving our key finding.

Second, we tested whether our experimental condition (sham vs. active iTBS) affected awareness of either the confederate’s mannerisms or their own mannerisms. In order to assess this, we conducted two separate chi-square tests of independence predicting from experimental condition (sham vs. active iTBS) the dummy-coded outcome of whether or not participants noticed: (1) the confederate touching her hair and face and (2) themselves touching their own hair and face. For these analyses, the sample size was 56 participants. Of the overall sample, 18 participants reported noticing the confederate touching her hair and face. However, the likelihood of noticing the confederate’s hair and face touching did not differ by experimental condition, χ (1) = 0.03, p = .85. Of the overall sample, three participants reported noticing themselves touching their own hair and face. However, the likelihood of noticing themselves touching their own hair and face did not differ by experimental condition, χ (1) = 0.43, p = .51. Therefore, the experimental condition did not seem to influence awareness of hair and face touching.

Third, we tested the effect of iTBS on each of the five moods that were reported (sad, nervous, happy, angry, and tired). We calculated change in mood by subtracting the mood rating before iTBS from the mood rating after iTBS. The change scores for the happy and tired moods were normally distributed so we ran independent samples t-tests in order to compare means on the happy and tired mood change scores in the sham and active iTBS conditions. The change scores for the happy, t(53) = 1.00, p = .32, and tired, t(53) = 1.20, p = .24, moods did not differ in the sham and active iTBS conditions. The change scores for sad, nervous, and angry moods were not normally distributed so we ran Mann-Whitney U tests. The change scores for sad, U = 358.50, p = .75, nervous, U = 344.50, p = .58, and angry, U = 376.50, p = .99, moods did not differ in the sham and active iTBS conditions. Thus, our findings suggest that iTBS did not effect mood.

## Discussion

The current study pitted two hypotheses against one another to determine the role of the rTPJ in the top-down control of mimicry. This study showed that–relative to the sham stimulation condition–the active stimulation condition led to less mimicry, suggesting that higher rTPJ activation inhibits mimicry in a social context. This finding is consistent with fMRI research showing that inhibiting an imitative response is associated with higher activation in the rTPJ [10] and with a tDCS study demonstrating that higher activation in the rTPJ leads to an improved ability to inhibit a prepotent imitative response [27]. This study adds to this literature by showing that even in a social interaction, which is considerably more complex and less controlled than the contexts in which previous studies have been conducted, higher activation in the rTPJ inhibits mimicry.

Given that previous studies have focused on the effect of rTPJ on mimicry when inhibiting mimicry was the goal [25],[27], we wanted to test the effect of rTPJ on mimicry in actual social interactions when mimicking is an adaptive behavior. This test allowed us to determine whether higher rTPJ activation inhibits the natural tendency to imitate regardless of the context or whether higher rTPJ activation facilitates mimicry in a social interaction when mimicking is an adaptive behavior, therefore indicating sensitivity to the context. Given that we found that higher rTPJ activation leads to less mimicry, our results are consistent with a role for rTPJ in distinguishing between self and other, putatively by biasing toward self representations over other, therefore leading to less mimicry. The results from our study do not support the alternative hypothesis that higher activation in the rTPJ leads to flexible control of self-other representations, enhancing representations of self over other–or other over self–in line with the goals of a given context. In other words, we find evidence supporting the hypothesis that the rTPJ seems to help us keep track of our own motor plans and behaviors so that we do not end up mimicking everything we see other people do.

Our results differ from the only other study that tested the effect of neurostimulation on behavioral mimicry within an actual social interaction [30]. Although Hogeveen and colleagues [42] found an effect of tDCS on mimicry when stimulating the rIFC, they did not find an effect when stimulating the rTPJ, which contrasts with our result of an iTBS effect when stimulating the rTPJ. The discrepancy may have to do with the fact that TBS produces stronger effects on neurons than tDCS. While tDCS generates very small currents within the brain, leading to membrane voltage changes of only a fraction of a millivolt, which can only bias neural firing, TBS produces much larger currents on the order of tens of millivolts, which can directly induce action potentials. Moreover, the patterned stimulation used by iTBS mimics theta-modulated gamma frequency firing, which may directly cause long-term potentiation in targeted neurons, whereas the constant current of tDCS may only influence such neural changes. Thus, the two types of stimulation differ in important ways and are likely to have distinct effects on the stimulated networks involved in mimicry. Despite these differences, our studies inform one another. Taken together, it seems that rIFC may be the gas pedal and rTPJ may be the brake when it comes to mimicry.

Our findings contribute to the mimicry literature in that they point to a brain region that may be a gating mechanism for mimicry. In other words, the role of rTPJ may explain why we do not mimic everything we see others do. At this point, the mirror-neuron system has received extensive attention, but its counterpart–a mechanism gating mimicry response–has not. Recently, however, attention has been paid to what happens when the system that distinguishes between self and other fails. Indeed, the boundary between self and other can blur to such an extent that confusion actually arises. In *Entanglement* (National Public Radio, January 2015), a woman talks about her experience with a neurological condition called mirror-touch synaesthesia, a condition that causes her to actually experience physically and emotionally what she observes in others. If she sees someone receiving a hug, she physically feels the sensations involved. If she sees someone looking sad, she takes on the emotion observed. She loses herself in the moods and movements of others; her sense of self almost completely merging with those around her. Her neurological condition demonstrates what happens when the self-other system malfunctions. Mirror-touch synaesthesia is a disorder that centers on difficulties inhibiting representations of others [43],[44]. Those with the condition perform more poorly than healthy controls in imitation-inhibition tasks [43] and they also have smaller gray matter volumes in the right angular gyrus, a brain region corresponding to rTPJ [16]. Although the mechanism underlying mirror-touch synaethesia is not known [44], our findings suggest it may involve impairments in a gating mechanism controlling mimicry, which includes the rTPJ, a region that helps distinguish self and other.

Mimicry has been hypothesized to arise from bottom-up as well as top-down processes [45]. For example, merely seeing another person enact a behavior can lead someone to automatically enact the same behavior (bottom-up mechanism) as can having an affiliation goal (top-down mechanism). In our study, we find that increasing activation in the rTPJ decreases mimicry but has no effect on affiliation motivation. This suggests that rTPJ affects mimicry by breaking the perception-behavior link, enhancing representations of self over other and leading to a decreased tendency to automatically mimic observed actions of others. The fact that increased activation in rTPJ did not affect affiliation motivation suggests that the effect of rTPJ on mimicry is not mediated through this psychological process. This finding gives further insight into the role that rTPJ plays in mimicry.

### Limitations

A critical limitation of this study is the use of sham stimulation as our control. Given that active TBS can be painful while sham TBS is not, we cannot disambiguate the neuromodulatory effects of TBS from its physical sensation. Indeed, some previous research has shown that effects reported to be caused by the neuromodulation may have been driven by pain instead [46]. A strong test of this hypothesis would involve the use of continuous TBS (cTBS), an inhibitory pattern of theta burst stimulation. Using the same paradigm as the current study, if the cTBS group shows more mimicry than the sham group, then this would provide evidence that TBS affects mimicry via its neuromodulation of the rTPJ. However, if the cTBS group, like the iTBS group, shows less mimicry than the sham group, then this would provide evidence that TBS affects mimicry via its painful sensation, potentially by putting people in a less prosocial state of mind. Despite the limitations of our study design, there are a couple reasons to have confidence that our results were not driven by pain. First, we did not find effects of iTBS on critical self-report measures of how motivated participants were to get along with the confederate, how much effort they made to get along with the confederate, and how much they thought the confederate liked them. Second, a wide range of studies using painful neurostimulation protocols, such as high-frequency rTMS and anodal tDCS, have relied on sham controls and have demonstrated that neurostimulation leads to increases in prosocial behavior, emotional mimicry, trust, and cooperation [47–49] and decreases in aggression and hurt feelings after social exclusion [50],[51]. These effects would not have been observed if pain were to put people in a less prosocial state of mind and the effects were driven by pain and not neurostimulation.

In order to understand the unique role that the rTPJ plays in the control of mimicry, future research should consider the use of control sites. Selecting a control site, however, would require more knowledge about the network of brain regions involved in the control of mimicry in order to not choose a control site that is anatomically and functionally connected to other regions involved in the control of mimicry. An additional limitation is that we have modulated only one brain region and there are many brain regions involved in mimicry. For example, the mPFC may play a crucial role in modulating mimicry based on goals given the role of the mPFC in mimicry [10],[11] as well as in top-down processing of desired outcomes (i.e. goals) [52]. Future research should explore the effect of the mPFC on goal-modulated top-down control of mimicry. Previous research has implicated other regions in the prefrontal cortex in the control of imitation as well [53],[54]. One study used dynamic causal modeling to understand the processing stream involved in the control of imitation. This study reported a processing stream from the medial frontal cortex to the insula to the inferior frontal cortex [54]. Importantly, this study as well as others did not find the TPJ to be meaningfully linked to the control of imitation [53],[54] even when using a paradigm that previously indicated involvement of the TPJ [10],[11]. Given that no single brain region is entirely responsible for mimicry, researchers should strive to better understand how the various brain regions involved in mimicry work together.

## Conclusions

This paper makes a number of contributions to the study of the neurophysiological underpinnings of mimicry and to the use of noninvasive brain stimulation to enhance understanding in this area. First, this study showed that higher rTPJ activation inhibits mimicry in a social context. Second, this finding allowed us to disambiguate between two competing hypotheses regarding mimicry. Given previous research showing that higher activation in the rTPJ reduces the tendency to imitate when inhibiting imitation is the conscious goal [55], we wanted to test the effect of rTPJ on mimicry in actual social interactions when mimicking is a nonconscious, adaptive behavior. Because we find that higher rTPJ activation inhibits mimicry even in a social context in which mimicking is typically beneficial, our findings do not support the role of rTPJ in flexibly enhancing representations of self over other–or other over self–in line with the goals of a given context. Rather, our findings are consistent with a role for rTPJ in distinguishing self from other, putatively by biasing toward self representations over other representations. Third, we show that iTBS can produce behavioral changes in a social interaction, which is a considerably more complex and less controlled context than the type of behaviors typically studied in the lab. We hope that this will encourage future researchers to study behaviors in more naturalistic contexts that yield greater external validity.
